# On Cerebral Congestion in Relation to Hemorrhage and Ramollissement of the Brain

**Published:** 1849-01

**Authors:** 


*On Cerebral Congestion in relation to Hemorrhage and Ramollissement of the Brain.* By M. Durand Fardel.—The object of this paper is to direct attention to the important part which congestion plays in inducing these conditions. Its influence may be sometimes judged of by premonitory symptoms, such as giddiness, singing in the ears, numbness of the limbs ; still, in 59 cases, 32 of acute ramollissement, and 27 of hemorrhage, the author only finds these recorded in 21. M. Rostan, to whom is due the first accounts of *ramollissement*, regarded the disease sometimes as an encephalitis, and sometimes as a peculiar degeneration analogous to senile gangrene, two very opposite conditions ; but the author, who has long had opportunities of observing it among the aged, maintains that in them also it is an encephalitis. In the dissection of 29 such cases which he has published, all the usual signs of congestion of the brain were observable. In the production of *cerebral hemorrhage*. Abercrombie and Bouillaud lay much stress upon the ossified condition of the vessels, and Rochoux regards it as proceeding from a special alteration, a hæmorrhagiparous ramollissement of the cerebral pulp. It is certain that a rarefaction rather than a ramollissement of this tissue does take place, and in almost all aged persons the meningeal vessels have become more friable ; but mere diminution of cohesion of the cerebral fibre would never give rise to hemorrhage, and ramollissement may go through all its stages without doing so. So, too, when hemorrhage takes place earlier in life, changes in the vessels are not observed ; and even later, such altered vessels are not to be followed into the substance of the brain, where the hemorrhage really takes place. A fluxion of blood to the part explains all the phenomena ; and the author believes that the rigid bony canals in which the veins are placed, cause this organ to be freed of any surplus blood with difficulty. If congestion of the brain or encephalitis occur at an earlier period of life, there is usually some appreciable cause discoverable ; but this is not so in old age, when they would seem rather to become developed under the influence of physiological modifications, independently of external circumstances. Life, as age approaches, seems to retreat from the periphery to the centre. First, the functions of the skin and of the senses diminish in activity, and the systems of voluntary motion and of organic life, the secretions and the digestive functions, die, or at least are gradually enfeebled. The lungs, the heart and the brain, alone preserve their physiological activity, and they alone become liable to disease ; and thus it is that the aged die of affections of the lungs, or the brain.

M. Rochoux, in the discussion which followed the reading this paper, observed that he still adhered to the opinion he expressed in 1814, that in the great majority of cases of *apoplexy*, there are none of the so-called premonitory symptoms present ; so, too, the great bulk of persons suffering from these, never become the subjects of apoplexy. A vast number of cases of apoplexy are on record, which occurred while the patient presented no circumstance indicative of congestion; and, on the other hand, various employments, the games of children, attacks of epilepsy, &c., give rise to great congestion without inducing it. M. Rochoux believes there is an imperceptible, organic, molecular change effected in more or less of the cerebral tissue, diminishing its power of cohesion and resistance, until, at last, unable to withstand the force of the circulating blood, it becomes torn, and the hemorrhage we term apoplexy is produced. The softening is just the same whether death takes place in an hour, or in several days, and must be regarded as precedent, not consecutive, to the effusion. This *hemorrhagiparous ramollissement* exerts nearly all the action attributed to all the other producing causes of apoplexy, including local or general congestion.

M. Baillarger observed that another consequence may be said to result more directly from congestion of the brain than these mentioned, viz. *general paralysis.* The patient on coming to himself, is found to have some embarrassment of speech, and gradually his intellect fails him. The anatomical changes in general paralysis have especial reference to the periphery of the brain, and we can easily conceive here the direct operation of repeated congestion. In hemorrhage and ramollissement, besides congestion, there must be some local lesion of the organ, which explains why one portion rather than another is attacked.—*Ibid, from Bulletin de l'Academie.*

